# Draft Genome Sequence of Xylaria longipes DSM 107183, a Saprotrophic Ascomycete Colonizing Hardwood

**DOI:** 10.1128/MRA.00157-19

**Published:** 2019-03-21

**Authors:** Enrico Büttner, Anna Maria Gebauer, Martin Hofrichter, Christiane Liers, Harald Kellner

**Affiliations:** a Department of Bio- and Environmental Sciences, International Institute Zittau, Technische Universität Dresden, Zittau, Germany; University of California, Riverside

## Abstract

The saprotrophic soft-rot fungus Xylaria longipes was isolated from deadwood of Acer pseudoplatanus collected in the Bavarian Forest, Germany. The whole genome of this strain (DSM 107183) was sequenced with a total size of 43.2 Mb and a G+C content of 48.5%. The genome comprises 12,638 predicted coding sequences.

## Abstract (precis)

The saprotrophic soft-rot fungus Xylaria longipes was isolated from deadwood of Acer pseudoplatanus collected in the Bavarian Forest, Germany. The whole genome of this strain (DSM 107183) was sequenced with a total size of 43.2 Mb and a G+C content of 48.5%.

## ANNOUNCEMENT

Xylaria longipes is one of the most common representatives of the ascomycete family Xylariaceae that includes saprotrophic fungi causing a specific type of wood decay (soft-rot type II) (1). The fungus is found in Central and Northern Europe and North America where it usually grows on *Acer* or *Fagus* spp. but also in subtropical forests (2). It is the subject of basic and applied scientific interest due to efficient degradation of hardwoods by enzymatic mechanisms differing from those of “classic” white-rot basidiomycetes. There are indications that—besides various polysaccharide hydrolases—laccases represent the main secreted enzyme type involved in wood decay by Xylariaceae (3, 4). To support this assumption, genome analyses of representative Xylariaceae species may be used to identify lignin-modifying oxidoreductases (i.e., class II peroxidases and laccases) and additional enzymes possibly involved in lignocelluloses decomposition, such as hydrolases.

X. longipes DSM 107183 (GenBank accession number MK408619) was isolated from a fallen maple log (Acer pseudoplatanus; Germany, Bavarian Forest, 48°58′25.3″N, 13°24′25.4″E). Mycelium was obtained from an agitated liquid culture (2.5% whey-protein glucose medium) inoculated with a pregrown fungal pure culture. Genomic DNA was extracted using a standard cetyltrimethylammonium bromide (CTAB)-based protocol. DNA was sheared into 200-bp fragments using adaptive-focused sonography (Covaris S2; Woodingdean Brighton, United Kingdom). The draft whole-genome was sequenced using an Ion Torrent PGM platform (Ion PGM Sequencing 200 kit version 2, 318v2 chip) with a 200-bp fragment library (Ion Xpress Plus fragment library kit; Thermo Fisher, Darmstadt, Germany). Altogether, 6.1 million sequence reads were filtered (180- to 280-bp lengths) and trimmed using Geneious R10 (error probability limit, 0.05; trim 3′ end) (5), and *de novo* assembly was performed using MIRA 4.0 (6). Assembly quality based on the *N*_50_ value versus the number of contigs was verified by QUAST version 4.5 (7). Completeness of the assembly was checked using BUSCO version 3.0 (8) (universal single-copy orthologs; predictor, Histoplasma capsulatum; fungi data set, Ascomycota_*odb9*) based on the ascomycete data set for contigs greater than 1,000 bp. The prediction of putative reading frames was performed using AUGUSTUS v3.2.2 (9) (predictor, Histoplasma capsulatum), and found genes were annotated with Blast2GO v5.2.5 (BioBam, Valencia, Spain). CAZyme annotation was performed using the Web interface dbCAN (E value, <1e^−15^; coverage, >0.35) (10). In summary, the *de novo* assembly with 1,006 contigs (1,000 chromosomal and 6 mitochondrial contigs; maximum length, 352,334 bp) has an *N*_50_ value of 74,055 and an average G+C content of 48.5%. The assembled draft genome of X. longipes has an estimated size of 43.2 Mb and includes 12,638 predicted genes. An analysis of BUSCO showed a completeness of the genome of 90.6%. Altogether, 708 carbohydrate-related enzymes and modules (149 enzymes with auxiliary activities, 106 carbohydrate esterases, 281 glycoside hydrolases, 90 glycosyltransferases, 17 polysaccharide lyases) and 65 carbohydrate-binding modules (CBM) were identified, and among them were 12 from CBM family 1 (cellulose binding) and 11 from CBM family 18 (chitin binding). Enzymes of interest (generic peroxidases, dye-decolorizing peroxidase, heme thiolate peroxidases, and lytic polysaccharide monooxygenases) are available at GenPept-associated BioProject accession number PRJNA395971. This draft genome will provide useful information for analyzing the enzymatic toolbox of ascomycetes causing soft-rot and comparing it with those of other wood-rot fungi.

### Data availability.

This whole-genome shotgun project has been deposited at DDBJ/ENA/GenBank under the accession number NQIL00000000. The version described in this paper is the first version, NQIL01000000. The Sequence Read Archive (SRA) accession number is SRR5883173.
